# Individual Based Model Links Thermodynamics, Chemical Speciation and Environmental Conditions to Microbial Growth

**DOI:** 10.3389/fmicb.2019.01871

**Published:** 2019-08-13

**Authors:** Valentina Gogulancea, Rebeca González-Cabaleiro, Bowen Li, Denis Taniguchi, Pahala Gedara Jayathilake, Jinju Chen, Darren Wilkinson, David Swailes, Andrew Stephen McGough, Paolo Zuliani, Irina Dana Ofiteru, Thomas P. Curtis

**Affiliations:** ^1^School of Engineering, Newcastle University, Newcastle upon Tyne, United Kingdom; ^2^Chemical and Biochemical Department, School of Applied Chemistry and Materials Science, University Politehnica of Bucharest, Bucharest, Romania; ^3^School of Engineering, University of Glasgow, Glasgow, United Kingdom; ^4^School of Computing, Newcastle University, Newcastle upon Tyne, United Kingdom; ^5^Department of Oncology, University of Oxford, Oxford, United Kingdom; ^6^School of Mathematics, Statistics and Physics, Newcastle University, Newcastle upon Tyne, United Kingdom

**Keywords:** individual based model, thermodynamics, chemical speciation, nitrification, methanogenesis

## Abstract

Individual based Models (IbM) must transition from research tools to engineering tools. To make the transition we must aspire to develop large, three dimensional and physically and biologically credible models. Biological credibility can be promoted by grounding, as far as possible, the biology in thermodynamics. Thermodynamic principles are known to have predictive power in microbial ecology. However, this in turn requires a model that incorporates pH and chemical speciation. Physical credibility implies plausible mechanics and a connection with the wider environment. Here, we propose a step toward that ideal by presenting an individual based model connecting thermodynamics, pH and chemical speciation and environmental conditions to microbial growth for 5·10^5^ individuals. We have showcased the model in two scenarios: a two functional group nitrification model and a three functional group anaerobic community. In the former, pH and connection to the environment had an important effect on the outcomes simulated. Whilst in the latter pH was less important but the spatial arrangements and community productivity (that is, methane production) were highly dependent on thermodynamic and reactor coupling. We conclude that if IbM are to attain their potential as tools to evaluate the emergent properties of engineered biological systems it will be necessary to combine the chemical, physical, mechanical and biological along the lines we have proposed. We have still fallen short of our ideals because we cannot (yet) calculate specific uptake rates and must develop the capacity for longer runs in larger models. However, we believe such advances are attainable. Ideally in a common, fast and modular platform. For future innovations in IbM will only be of use if they can be coupled with all the previous advances.

## Introduction

The microbial world is difficult or impossible to observe and with many processes and phenomena that transcend human experience and intuition. Mathematical modeling is a correspondingly vital, but underdeveloped, aspect of microbial ecology. Models can link theory and observations, reconcile seemingly contradictory experimental results (Drion et al., 2011), and guide and complement experimental plans (Widder et al., 2016).

The characteristics we can observe in microbial systems are the emergent properties of millions of individuals, in dozens of functional groups and hundreds of species. These emergent properties are best captured in modeling practice by individual (or agent) based approaches. Individual based models (IbM) treat every microorganism as a separate entity or agent, with their own set of parameters. In the model, as in real life, the individual shapes its surroundings by consuming nutrients, excreting metabolites and interacting with neighboring cells.

Since the landmark paper of Kreft et al. (1998), IbM have gained wider acceptance, being employed for the study of ecological behaviors, for example, cooperation vs. competition (Xavier and Foster, 2007), public goods dilemma (Mitri et al., 2011), division of labor (Dragoš et al., 2018), and survival strategies, such as bacteriocin production (Bucci et al., 2011) or response to phage infection (Simmons et al., 2017). IbM have a variety of environmental applications, especially in wastewater treatment systems [activated sludge systems (Picioreanu et al., 2004; Matsumoto et al., 2010; Ofiteru et al., 2014), anaerobic digestion (Batstone et al., 2006; Doloman et al., 2017) and microbial fuel cells (Picioreanu et al., 2010)] and are, if large enough, well suited to the study of evolution, most recently in the sea (Hellweger et al., 2018). A recent authoritative review highlighted the advantages, disadvantages, potential and challenges of IbM (Hellweger et al., 2016).

Scale is particularly important: the computational demands of IbM will always place a limit on the scale at which they can be applied. However, it is now evident that this limit can be overcome by the use of statistical emulators (Oyebamiji et al., 2017). In principle, this new approach will allow the output of an IbM to be used at an arbitrarily large scale. This is a strategically important advance that creates a new impetus for the development of credible IbM.

As the field of IbM matures from being an intriguing research exercise to a used and trusted tool, modelers must strike a balance between having a tractable computational burden and sufficient features to make credible predictions. Those features must be chosen carefully (in the light of the underlying hypothesis) and, wherever possible, grounded in a fundamental truth.

The laws of thermodynamics are one such truth that is of known predictive power in microbial systems (Broda, 1977; Jetten et al., 1998). McCarty's seminal work (McCarty, 2007) in this area used this insight to estimate yields and his work was subsequently built on by Heijnen et al. (1992) and most recently by González-Cabaleiro et al. (2015a). Despite the obvious power of this approach it has been almost overlooked in IbM (Araujo Granda et al., 2016), in favor of the less challenging use of a simple Monod function. All metabolisms and therefore all metabolic models are subject to the laws of thermodynamics. Consequently, a thermodynamic approach could represent a tractable “halfway house” between the ideal of a constraint based metabolic model [advocated by Hellweger et al. (2016)] and the simple Monod function typically employed.

Any model considering thermodynamics must also take account of pH and thus, ideally, the carbonate-bicarbonate buffering system and the speciation of key solutes in the system. pH and speciation are also fundamental to the ecology of microbial systems. Not only is pH the “master variable” in most microbial systems, but speciation is a very simple yet very important feature of microbial growth. For example, since ammonia is available to ammonia oxidizing bacteria (AOB) but ammonium is not, a decrease in pH can affect the growth of AOB simply by reducing the ammonia available for growth. Speciation should always be considered before more complex notions such inhibition or toxicity are invoked (Prosser, 1990). However, pH and speciation are typically [but not invariably (Batstone et al., 2006)] overlooked in newer modeling frameworks (Naylor et al., 2017).

We also note and propose that if IbM are to be credibly upscaled, they must also be: connected to their putative environment (that is not isolated from the bulk), in 3-D, be sufficiently computationally efficient to enable a meaningfully long simulation in a realistic amount of time and have at least basic mechanical features (Winkle et al., 2017).

This paper presents the working principles of a multispecies IbM that meets this challenge. This is a generalizable model that can, in principle, be used for any redox couple in any system. A feature we have sought to exemplify by using the same framework to model an aerobic system (nitrification) and an anaerobic one (anaerobic digestion).

The growth process is modeled using thermodynamic principles, enabling the estimation of growth yields according to the chemical energy of the environment. The acid-base chemistry is comprehensively described by an explicit sub-model that can account for maximum three deprotonations. The mechanical interactions can describe attachment and detachment of microorganisms in the biofilm and the pressure released when bacterial division occurs, which leads to cell re-arrangement. In addition, the results stress the need to employ reactor mass balances and consider the influence of environmental conditions on biofilms, especially for multispecies systems, exhibiting syntrophic and/or competitive relationships. The model outputs can be emulated using the approach proposed in Oyebamiji et al. (2017) and employed for large-scale CFD simulations in the future.

## Materials and Methods

The mathematical model clusters the main phenomena considered under three main “conceptual” categories: biological, chemical and mechanical.

### Biological Module

The model employs a traditional IbM approach in the description of agents, modeling them as spheres with their own parameters, chemical formulae and functionalities. To begin with, we place the microbial agents inside a 3-D simulation domain with dimensions of 100 × 20 × 300 μm (length × width × height) and discretized using a uniform grid of 50 × 10 × 150 points. This computational domain can accommodate ~500,000 particles, with an average diameter of 1 μm. The physico-chemical characteristics of the microbial agents closely mimic those of real-life systems, employing the chemical formula, CH_1.8_O_0.5_N_0.2_, proposed by Roels (2009). While both physical parameters and elemental composition are easily determined experimentally, it is significantly more complicated to accurately define growth parameters for individual bacterial cells (Hellweger et al., 2016). As stated above, we use the thermodynamic approach of González-Cabaleiro et al. (2015b) for growth yield estimation, but an empirical Monod formulation for microbial growth. We have reduced the complex metabolic networks to two main simplified reactions: one for anabolism and one to describe the catabolic pathway. The thermodynamic yield estimation methodology assumes that the maximum growth yield of a microorganism, Equation (1), is dictated by the balance between:

- the free energy requirement for its anabolic pathway, Δ*G*_*ana*_
- the energy available from its catabolic pathway, Δ*G*_cat_, using its absolute value
- the energy dissipated for maintenance requirements, Δ*G*_dis_

(1) $$\begin{array}{l} Y_{XS}=\frac{\Delta G_{cat}}{\Delta G_{ana}+\Delta G_{dis}} \end{array}$$

where Y_*XS*_—growth yield for biomass with respect to the electron donor.

The free energies for catabolism and anabolism can be easily determined, provided the free Gibbs energies for chemical species considered are readily available and should be corrected for the environmental temperature. The dissipation energy (Δ*G*_dis_) is computed using the correlation proposed by Tijhuis et al. (1993) or user supplied. The anabolic and catabolic reactions are combined in an overall growth reaction, function of the energy balance, ensuring that thermodynamic restrictions are not violated for the entire computational domain. An example calculation for the yields of ammonia oxidizing bacteria is presented in the Supplementary section Thermodynamic Calculations.

The specific growth rate for each bacterial cell (μ) assumes a Monod-type expression, using generic multiple substrate limitation, defined in Equation (2):

(2) $$\begin{array}{l} \mu =q_{max}\cdot Y_{XS}\cdot \Pi_{i}\frac{C_{Si}}{K_{Si}+C_{Si}}-m_{bac} \end{array}$$

where *q*_max_ is the maximum substrate uptake rate, *K*_*Si*_ is the half saturation/affinity constant and *C*_*Si*_ is the growth limiting substrate concentration corresponding to the *ith* substrate.

The growth equation employs a maintenance term, *m*_bac_, whose value is computed using Equation (3):

(3) $$\begin{array}{l} m_{bac}=\frac{\Delta G_{dis}}{\Delta G_{cat}} \end{array}$$

Thus, the growth model assumes mixed kinetic–thermodynamic limitation, detailed in the work of González-Cabaleiro et al. (2015b), considering three possible scenarios for bacterial growth:

- if $m_{bac}>\alpha \cdot q_{max}\cdot Y_{XS}^{max}\cdot \Pi_{i}\frac{C_{Si}}{K_{Si}\;+\;C_{Si}}$, the biomass agent grows, its mass increasing according to the mass balance in Equation (4)
  (4) $$\begin{array}{l} \frac{dX_{i}}{dt}=\mu_{i}\cdot X_{i} \end{array}$$
- if $\beta \cdot q_{max}\cdot Y_{XS}^{max}\cdot \Pi_{i}\frac{C_{Si}}{K_{Si}\;+\;C_{Si}}<m_{bac}<\alpha \cdot q_{max}\cdot Y_{XS}^{max}\cdot \Pi_{i}\frac{C_{Si}}{K_{Si}\;+\;C_{Si}}$, the biomass agent neither grows nor decays, its mass remains constant, Equation (5)
  (5) $$\begin{array}{l} \frac{dX_{i}}{dt}=0 \end{array}$$
- if $m_{bac}<\beta \cdot q_{max}\cdot Y_{XS}^{max}\cdot \Pi_{i}\frac{C_{Si}}{K_{Si}\;+\;C_{Si}}$, the biomass agent undergoes decay, the bacterial mass declines via a first order process, Equation (6)
  (6) $$\begin{array}{l} \frac{dX_{i}}{dt}=-k_{decay,i}\cdot X_{i} \end{array}$$

where α and β refer to relaxation non-dimensional parameters of the equal energy condition between the local environment and the maintenance required by the cell (by default 1.2 and 0.8), *X*_i_ is the mass, μ_i_ is the specific growth rate and *k*_*decay,i*_ is the decay rate constant for agent *i*.

To close the mass balance, the products of cellular decay are the carbon and nitrogen source specified by the anabolic reaction.

The diameter of bacterial agents' increases as the agents are consuming nutrients and excreting metabolic products, according to their corresponding mass balance equation (Equation 4). We impose a value of the agent's diameter (Table S5) at which a bacterial agent instantaneously divides to form two “daughter-agents.” The mass of the parent is randomly distributed between the two daughters, each accounting for up to 50 ± 10% of the mass of the initial agent (Kreft et al., 1998).

To determine the positions of the cells after a division event, one of the daughters retains the position of the parent, while the second is placed on a spherical trajectory around it and the final position of both cells is determined after performing the mechanical calculations, presented in section Mechanical Module. We propose that as an agent reaches a threshold radius (corresponding to a cell with 10% division mass) it obtains inert status and no longer participates in the biological process.

### Chemical Module

The chemical module's focus is modeling the transport and uptake of nutrients/excretion of metabolic products, describing the effect of chemical speciation and gas-liquid equilibrium.

#### Mass Transport and Chemical Reactions

Due to the biofilms' high density and porous structure, it is assumed that nutrients are only transported by diffusion (de Beer et al., 1994). The diffusion phenomenon is modeled using the assumptions of Fick's second law. Because the soluble components can be consumed and/or produced inside the biofilm, the mass balance equation is updated with the corresponding reaction term, Equation (7)

(7) $$\begin{array}{l} \frac{dC_{S}}{dt}=D_{eff,S}\cdot \left(\frac{\partial^{2}S}{\partial x^{2}}+\frac{\partial^{2}S}{\partial y^{2}}+\frac{\partial^{2}S}{\partial z^{2}}\right)+\sum_{i}r_{i}^{x,y,z} \end{array}$$

where *C*_*S*_ is the molar concentration species of *S, D_e f f, S_* is the diffusion coefficient corresponding to chemical species *S* and $r_{i}^{x,y,z}$ represents the reaction term for species *S*, consumed or produced by microbial agent *i* at coordinates (x, y, z).

To estimate the biofilm diffusion coefficients, we considered the effect of biomass packing on internal diffusion (Kapellos et al., 2007). We tested two corrections proposed in literature: amending the diffusion coefficients function of biomass concentration (Ofiteru et al., 2014) or assuming 80% slower diffusion, compared to water (Lardon et al., 2011). Preliminary tests found that using the biomass density correction can lead to diffusion coefficients as low as 20% the values of those in water, while experimental values indicate a maximum of 40–50% reduction (Renslow et al., 2010).

As a result, we chose to use the conservative estimate, Equation (8), in the simulations presented here, despite not accounting for biomass density and assuming uniform diffusion resistance throughout the biofilm.

(8) $$\begin{array}{l} D_{eff,S}=0.8\cdot D_{S,water} \end{array}$$

For the remaining part of the simulation domain (i.e., not occupied by the biofilm), the entire diffusional resistance is concentrated in a boundary layer of fixed height of 40 μm, that is allowed to move as the biofilm expands. In the boundary layer, the chemical species' diffusion coefficients are equal to those reported for water (Table S1).

### Reactor Coupling

We assume that our model biofilm is located inside a larger bioreactor, whose performance both influences and is influenced by the biofilm behavior.

Conceptually, in the simulation domain we place a bulk liquid compartment on top of the boundary layer that accounts for the mass transport to/from the biofilm. In the same way to the boundary layer, we assume bacterial agents are not present in the bulk liquid compartment.

To model the bulk liquid compartment, we consider it representative of a larger continuous stirred tank reactor (which encompasses the biofilm). For the corresponding reactor mass balance we employ the dynamic equation proposed by Picioreanu et al. (2004), Equation (9):

(9) $$\begin{array}{l} \frac{dC_{S}}{dt}=\frac{Q}{V}\cdot \left(C_{S,in}-C_{S}\right)+\frac{A_{F}}{L_{x}\cdot L_{Y}}\cdot \frac{1}{V}\cdot \int \int \int_{0}^{V_{biofilm}}r_{S}dxdydz \end{array}$$

where *Q* represents the volume flow rate, *V* is the bioreactor volume, *C*_*S,in*_ is the reactor inlet concentration for component *S, A*_*f*_ is the biofilm surface area (in the bioreactor); *L*_*x*_*, L*_*y*_ are the length and width, respectively of the computational domain, *V*_*biofilm*_ is the biofilm volume and *r*_*S*_ is the reaction term corresponding to production/consumption of component S (Table S4).

Equation (9) accounts for the inlet and outlet flows of the larger reactor and the overall bio-reaction rates, averaged in the integral term in Equation (9)—on the scale of the computational domain. To transition to the bioreactor scale (Picioreanu et al., 2004), multiplied the overall rates with the ratio between the biofilm surface area in the bioreactor and that in the computational domain.

In this manner, the bulk liquid concentrations for all the chemical species are computed throughout the simulations, instead of considering the fluid volume an infinite source of nutrients.

#### Gas-Liquid Mass Transfer

The gas–liquid mass transfer is an important factor determining the performance of biological wastewater treatment: improper aeration can cause substrate limitation and treatment failure, while for anaerobic digestion, the dissolved hydrogen concentration can lead to the selection of different metabolic pathways and formation of different products ranges (Khan et al., 2016).

The mass transfer is modeled using the two-film theory, having a gas liquid mass transfer rate *r*_*L−G*_ that is computed for every grid cell, using Equation (10):

(10) $$\begin{array}{l} r_{L-G}=k_{L}a\cdot \left(C_{S,L}-C_{S,i}^{*}\right) \end{array}$$

where *k*_*L*_*a* is the mass transfer coefficient (h^−1^), *C*_*S,L*_ is the concentration of gaseous component *S*, dissolved in the liquid phase, while $C_{S,i}^{*}$ is the saturation concentration corresponding to the partial pressure (*p*_*S*_) of component *S* in the reactor headspace. The mass balance for the gas phase components is written for the reactor headspace, modeled as a dynamic continuous stirred tank reactor, using Equation (11):

(11) $$\begin{array}{l} \frac{dp_{S}}{dt}=r_{L-G,ave}-\frac{Q_{gas}}{V_{gas}}\cdot p_{S} \end{array}$$

The rate of gas-liquid transfer (*r_L−G,e_*) is averaged over the computational domain, assuming a reactor headspace equal in size with that of the liquid space, following the methodology presented in Batstone et al. (2006).

#### pH calculations

In order to apply the thermodynamic framework, an explicit pH calculation module was implemented, capable of handling both hydration reactions (e.g., CO_2_ + H_2_O → H_2_CO_3_) and up to three deprotonations (e.g., H_2_CO_3_ → ${\text{HCO}}_{3}^{-}$ → ${\text{CO}}_{3}^{2-}$). The dissociations are assumed to occur instantaneously with respect to the rate of other phenomena considered and are modeled as equilibrium processes (Batstone et al., 2002). The full set of dissociation reactions considered in the model in presented in Table S2.

The procedure was adapted from Volke et al. (2005), expressing the concentration of each ionized species function of the proton concentration and total concentration of equilibrium forms. The dissociation equilibrium constants are computed from the species' free Gibbs energy (Table S3), adjusted for ambient temperature. The ensuing charge balance takes the form of a non-linear equation, solved for the proton concentration in each point of the computational domain, using a modified Newton Raphson algorithm.

Bacterial cells are usually able to take up only one form of the substrate (e.g. NH_3_ and not ${\text{NH}}_{4}^{+}$, acetic acid and not acetate), whose concentration and availability are in turn influenced by the diffusion, mass transfer and biological processes. To mimic this reality, we have amended the growth expressions for each agent to utilize only the appropriate form used by actual bacteria.

### Mechanical Module

This module aims to describe the mechanical behavior of individual bacteria within a community, solving the equation of movement proposed below for each particulate component, Equation (12), following the implementation presented in Jayathilake et al. (2017):

(12) $$\begin{array}{l} m_{i}\cdot \frac{d\vec{v_{i}}}{dt}=F_{c,i}+F_{a,i}+F_{f,i} \end{array}$$

where *m*_*i*_represents the mass of the bacterial agent and *v*_*i*_ —its corresponding velocity; and the following forces are acting on the agents:

- *F*_*c,i*_ is contact force, incorporating viscoelastic and friction forces between bacteria: The friction forces are modeled using the Kelvin-Voigt model both in normal and tangential direction, while for the estimation of tangential frictional forces we are using the Coulomb criterion.
- *F*_*a,i*_ is cell-cell adhesion force: modeled using an artificial spring constant, proportional to the mass of the two affected agents.
- *F*_*f,i*_ is fluid drag force: the effects of fluid flow on bacterial cells can be modeled using a oneway coupling, i.e. considering only the effect of flow on bacterial cells but not the other way round.

After the division computations are performed, the system is far from mechanical equilibrium and must return to its equilibrium state (i.e., internal pressure is relaxed). The movement equations for all particles are resolved, keeping in mind this assumption.

The specifics of the mechanical module implementation are presented in detail in Jayathilake et al. (2017) while the list of parameters used for the mechanical model is presented in Table S6.

### Implementation

Due to the high level of sophistication of this approach and the diverse time scales on which the modeled phenomena take place, the software implementation was committed to allow the computation of large domains for long simulation periods in a highly efficient manner. The mathematical model was implemented in the LAMMPS environment (Large-scale Atomic/Molecular Massively Parallel Simulator).

The source code and user manual are available on GitHub at https://github.com/nufeb/NUFEB.

#### Solving Strategy

The model assumes that, due to the time scale disparity between mass transport and biological growth, the two systems can be decoupled (Kreft et al., 2001). This allows the diffusion-reaction equations to reach steady state and resolves microbial growth on the longer time scale. The mechanical interactions are decoupled using the same assumption, but on an intermediate time scale.

**Figure 1:**
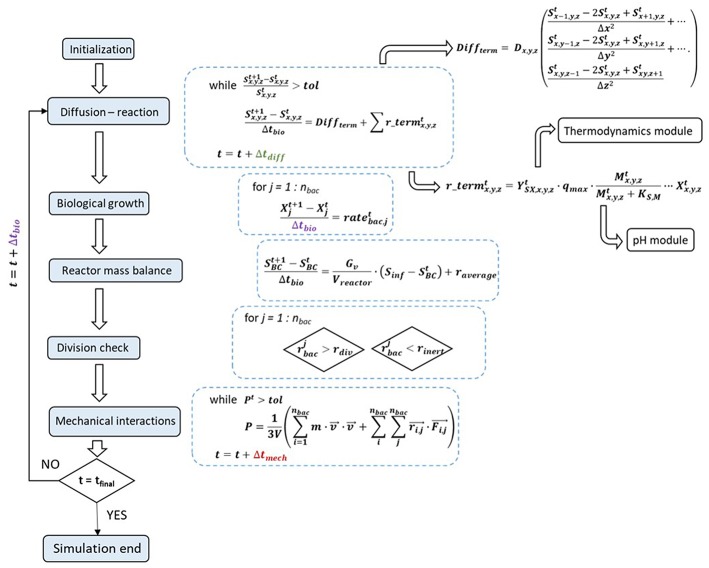
Solving algorithm and interactions between the model's modules, with their corresponding mathematical equations: initialization is followed by resolving the diffusion-reaction equations, biological growth and reactor mass balance, division and decay checks and mechanical interactions.

Following initialization of all simulation conditions, the first chemical computations are performed, to set the stage for solving the diffusion-reaction system of partial differential equations. At every diffusion iteration, both pH and thermodynamic modules must be called, to compute the chemical species' reactions rates, which are then added to the discretized diffusion term (Figure 1).

Upon reaching diffusional steady-state, the mass balances for the microbial agents are resolved, which in turn enables the determination of the reactor mass balances and update of the boundary conditions.

Afterwards, the division and decay checks are executed and the mechanical module comes into play to resolve the agent overlapping and all other physical interactions. The new and updated biomass positions are referred to the chemical module and a new iteration can begin.

#### Numerical Methods

The diffusion-reaction model is solved using a fully explicit finite difference method: the backward Euler method for time discretization and centered finite differences for the space derivatives.

The default boundary conditions used for the biofilm case are:

- Dirichlet boundary conditions at the top of the boundary layer: the values can be constant or variable. For the constant (or fixed) value case, we do not solve the reactor mass balance and consider the simulations decoupled. By solving the dynamic reactor mass balance, detailed in Reactor Coupling, the values of the Dirichlet boundary conditions will be updated every biological time step.
- zero-flux Neumann boundary conditions at the bottom of the computational domain;
- periodic boundary conditions on the lateral faces of the computational domain.

The mass balances corresponding to the bacterial agents and gas-liquid mass transfer are solved using a backward Euler algorithm while the mechanical relaxation equations are integrated using a discrete element method.

#### Time Stepping Strategy

Each main module has a defined time step for its calculations, with values ranging from 10^−4^ s for the diffusion calculations (Δtdiff) and 10^−3^ s for mechanical relaxation (Δtmech) to as high as 1 h for biological computations (Δt_bio_). The time stepping must be tailored by the user, in accordance with the bacterial growth rates and process conditions.

#### Code Parallelization

To provide one of the most comprehensive simulation tools for individual based modeling, a high level of description was required to account for the chemistry, biology and mechanics of biofilm formation. This, however, led to cumbersome computations and the need for significant computing power to run simulations in a timely manner. In order to lower the computational burden incurred, the code was parallelized.

The parallelization effort focused on two main areas: the mechanical interactions and the biological and chemical calculations.

For the former we employed a spatial domain decomposition strategy, which is the foundation of LAMMPS parallelism and already available in the software's Granular module, while for the latter we had to decompose the contents of the smallest computational unit, the grid cell. In both cases, the resultant subdomain was assigned to a different processor, and computations could be carried out independently, when their nature permitted.

However, during the computation of the pairwise interaction forces for the mechanical module and the diffusion-reaction calculations, information residing in a different processor was needed. As a result, we implemented a communication scheme based on the Message Passing Interface standard, for both the focus areas.

The spatial layout of the decomposition, which determines the size of each subdomain, was kept the same throughout the simulations, and was chosen in order to reach a good load balance during the biofilm steady state condition (i.e., toward the end of the simulation), when the computational load is greater due to the large number of particles.

Automatic vectorization was employed to speed-up a few computation intensive routines (e.g., pH calculation), with the need of using control directives (pragmas) to achieve the desired result in most of the cases.

All simulations were run on Newcastle University Rocket cluster using different numbers of processors in each case, while the run time limit for the simulations was imposed at 2 days by the cluster design.

## Results

We implemented the model in two highly contrasting scenarios: a simplified aerobic nitrifying system and a more complex anaerobic community. The results are grouped according to the system they represent, the simulations performed in each case are numbered, using indices *a* and *b* for the nitrifying and anaerobic systems, respectively.

### Aerobic System

The aerobic system considers two autotrophic functional groups: ammonia oxidizing bacteria (AOB) and nitrite oxidizing bacteria (NOB). The domain was seeded with AOB and NOB particles in a 1:1 ratio, evenly distributed in 8 layers at the bottom of the computational domain The initial distribution was chosen due to the cross-feeding relationship between the two bacterial types, in order to (initially) provide each NOB equal access to their substrate producing counterpart (AOB). The kinetic and thermodynamic parameters for the biological agents are presented in Table 1, together with the anabolic and catabolic reactions corresponding to each type.

**Table 1:** Kinetic and thermodynamic parameters for the aerobic functional groups.

**Aerobic system**
**Functional group**	**Kinetic parameters**				**References**
	**μ_max_** **(mol** **·** **L**^**−1**^ **h**^**−1**^**)**	**K**_**s-O2**_ **(mol** **·** **L**^**−1**^**)**	**Ks— NH**_**3**_**/NO**_**2**_ **(mol** **·** **L**^**−1**^**)**	**k**_**decay**_ **(h**^**−1**^**)**	
**AOB**	0.032	9.38_·_10^−7^	2.11_·_10^−6^	0.01	Picioreanu et al., 2016
Anabolic reaction	0.9 NH_3_ + HCO_3_^**−**^+ H^+^ → CH_1.8_O_0.5_N_0.2_ + 0.7 HNO_2_ + 1.1 H_2_O				
Catabolic reaction	NH_3_ + 1.5 O_2_ → NO_2_^**−**^+ H^+^ + H_2_O				
**NOB**	0.031	1.88_·_10^−6^	3.94_·_10^−9^	0.088	Picioreanu et al., 2016
Anabolic reaction	2.9 HNO_2_ + ${\text{HCO}}_{3}^{-}$ + H^+^ → CH_1.8_O_0.5_N_0.2_ + 2.7 HNO_3_ + 0.2 H_2_O				
Catabolic reaction	NO_2_^**−**^+ 0.5 O_2_ → NO_3_^**−**^				
	**Thermodynamic parameters**				
**Functional group**	**ΔG formation (kJ/Cmole-X)**	**ΔG dissipation (kJ/C-moleX)**	**Calculated Yield (C-mole-X/mole-eDonor)**		
**AOB**	–67	–3,500	0.155		Heijnen et al., 1992
**NOB**	–67	–3,500	0.077		Heijnen et al., 1992

The varied functionalities of the nitrifying model were demonstrated in five contrasting simulations (conditions presented in Table 2). The conditions varied between the five cases presented are the treatment of boundary conditions (fixed boundary conditions imply no reactor coupling and vice versa) and of the pH calculations. Unless stated otherwise, the concentration profiles presented in the figures below refer to the total concentration of all dissociation forms.

**Table 2:** Initial simulation conditions for the aerobic case study: initial concentrations refer to the total (i.e., protonated and un-protonated forms) concentration of the chemical compounds; the CO_2_ concentration presented in this table includes CO_2_, H_2_CO_3_, ${\text{HCO}}_{3}^{-}$ and ${\text{CO}}_{3}^{2-}$ forms, while NH_3_ refers to the total concentration of free ammonia (NH_3_) and ammonium ion (${\text{NH}}_{4}^{+}$); both NO_2_ and NO_3_ terms incorporate the nitric/nitrous acid and their corresponding ion concentrations.

		**Aerobic system**				
**Simulation conditions**		**Simulation #**				
		**1a**	**2a**	**3a**	**4a**	**5a**
**Concentration (mg/L)**		**Top boundary conditions**				
O_2_	9	Fixed	Fixed	Fixed	Fixed	Fixed
CO_2_	88	Fixed	Fixed	Fixed	Fixed	Fixed
NH_3_	30	Fixed	Variable	Fixed	Variable	Variable
NO_2_	0	Fixed	Variable	Fixed	Variable	Variable
NO_3_	0	Fixed	Variable	Fixed	Variable	Variable
**Initial value**		**pH Control**				
pH	7.5	Constant	Constant	Free	Free	Buffered
		**Simulation descriptor**				
		Fixed BC —constant pH	Dynamic BC —constant pH	Fixed BC —free pH	Dynamic BC —free pH	Dynamic BC —buffered pH

The five nitrifying simulations (Table 2) were:

- 1a “fixed boundary conditions simulation” where the Dirichlet boundary conditions (i.e., concentrations of the soluble species at the top of the boundary layer) are fixed, the reactor performance is decoupled from the biofilm.
- 2a “dynamic boundary conditions” where the Dirichlet boundary conditions are variable and their values are computed using the reactor mass balance module, the reactor performance is coupled;
- 3a “fixed pH simulations” where the pH was kept constant in the entire computational domain
- 4a “free pH simulations” where the pH was allowed to vary as function of the chemical species concentrations
- 5a “buffered pH simulations” in which the pH was buffered with Na^+^ and Cl^−^.

All simulations were run until the biofilm reached a height of 250 μm, with particles forming above this height being shaved off the top of the biofilm and taken out of the computational domain. The value of 250 μm was chosen to represent steady-state height, as experimental studies report values in the range 50–500 μm for oxygen penetration depth (Piculell et al., 2016). The simulations were further monitored until the biomass concentration profiles indicated that biofilm steady state was obtained.

#### Reactor Coupling

For the nitrification system, the boundary concentrations of O_2_ and CO_2_ were fixed in all simulations, assuming that through aeration they are kept constant at the top of the biofilm. The initial CO_2_ concentration value was chosen to buffer the system pH to 7.5, and to ensure the system is not limited by inorganic carbon.

**Figure 2:**
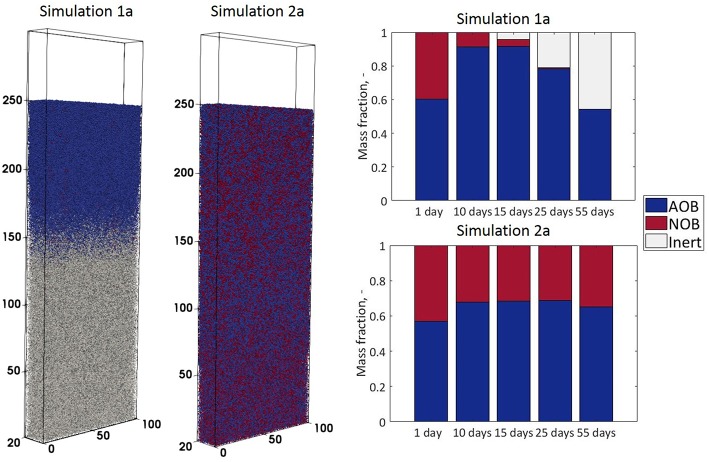
Biofilm structures obtained in simulations 1a and 2a (fixed vs. dynamic boundary conditions—at time *t* = 45 days) and biomass compositions (as fractions of total biomass)—obtained at different time steps.

A comparison of the biofilm structures and the evolution of AOB to NOB ratios for the two cases (simulation 1a and 2a) is presented in Figure 2.

**Figure 3:**
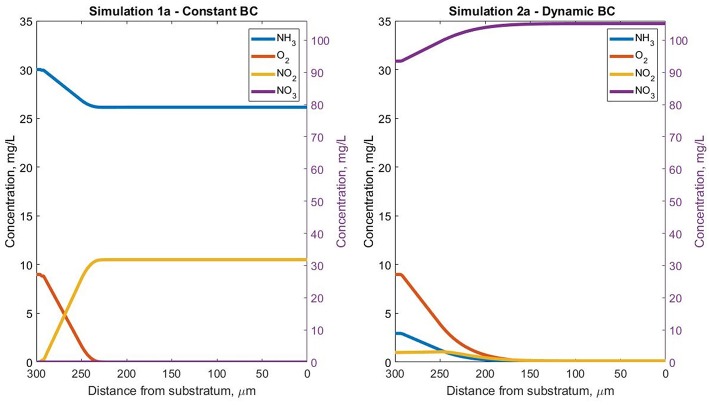
Steady-state soluble species concentration profiles for simulations 1a (fixed boundary conditions—constant pH) and 2a (dynamic boundary conditions—constant pH), in the Oz direction at coordinates *x* = 50 μm and *y* = 10 μm inside the biofilm.

In simulation 1a (fixed boundary condition) the nitrite diffuses out of the system, so the NOB population decays to form inert particulates, and AOB and inert particles dominate the system (Figure 2). By contrast, in simulation 2a, when the biofilm is coupled to the reactor, NO_2_ is supplied from the top (Figure 3) and its concentration in the biofilm ensures the NOB growth rate is higher than the maintenance costs. The steady-state biofilm (Figure 2) is comprised of both AOBs and NOBs, in a ratio of ~ 2:1.

The AOB steady-state biomass concentration in simulation 1a (uncoupled from the reactor) is less than half of that obtained in the coupled simulation 2a. The low activity of AOBs in the biofilm is seen in spite of the fixed boundary condition that ensures high ammonia concentrations are available for growth. This is because the bacterial population reaches the oxygen depletion stage (and entering maintenance and decay stages) faster in simulation 1a than simulation 2a (Figure 3). The total AOB growth rate is higher in simulation 1a than simulation 2a, but only for the first 10 days. The AOBs in 1a subsequently enter a stationary plateau reminiscent of a classical growth curve.

The inerts are accumulating in simulation 1a—they represent the NOB agents that decayed due to the small nitrite concentrations in the first simulation days and the AOB agents that suffer from oxygen limitation, more acutely than in simulation 2a.

The behavior is quite different under the dynamic boundary conditions (simulation 2a). The total biomass concentration appears to enter a permanent oscillatory state: reaching the height limit, the removal of a large number of particles alleviates the competition for oxygen and ammonia/nitrite, which leads to another biomass concentration increase. The putative oscillations in bacterial numbers and concentration suggest that a true steady-state biofilm cannot be obtained in this case. This has been observed previously (Matsumoto et al., 2010). The biomass concentration profiles are provided in Figure S1.

The concentration profiles of the soluble species are consistent with the biomass observations: in simulation 1a (where almost no NOB agents are present even before reaching the steady state), the total NO_2_ (nitrite and nitrous acid) accumulated in the biofilm (and NO_3_ was absent). In contrast, for simulation 2a both nitrite and nitrous acid are completely consumed by the NOBs and NO_3_ accumulated (Figure 3).

#### Influence of pH

The use of a model has allowed us to conduct experiments in which the pH can be (unrealistically) perfectly controlled (2a), allowed to vary naturally (4a) or systematically controlled (5a) (when the bulk pH drops below 6.5) as might happen in a well-managed reactor.

**Figure 4:**
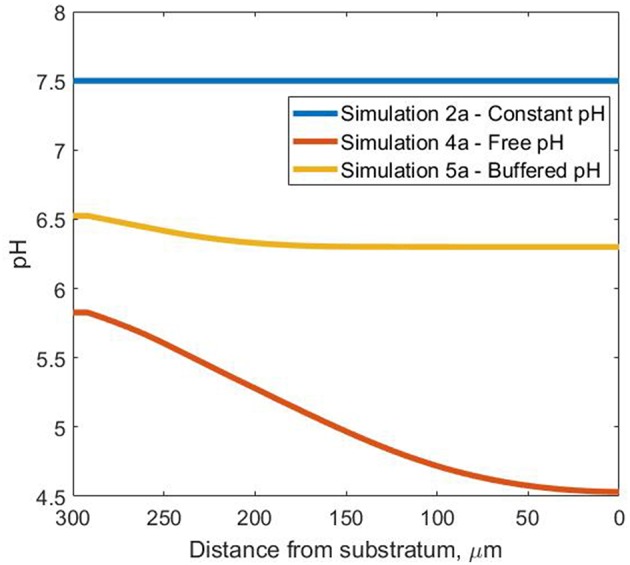
Steady-state pH profiles—in the Oz direction at coordinates *x* = 50 μm and *y* = 10 μm inside the biofilm for the three pH cases.

The steady-state pH profiles are presented in Figure 4 for all pH simulations, highlighting the wide range of pH values the bacterial agents are subjected to inside the biofilm. As expected, pH variation affected both the soluble species and biomass profiles in the three scenarios considered.

**Figure 5:**
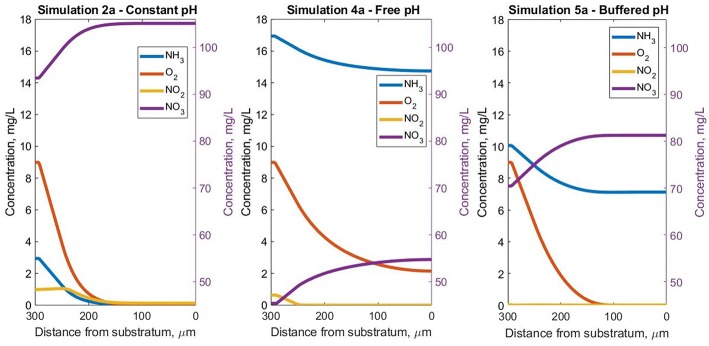
Steady-state soluble species concentrations in the Oz direction at coordinates *x* = 50 μm and *y* = 10 μm inside the biofilm, for the three pH simulations.

The drop in pH in simulation 4a ensures free ammonia concentrations are so low that ammonia is the limiting resource even though there is abundant total ammonia and the O_2_ concentration exceeds 2 mg/L in all biofilm regions (Figure 5). In contrast, in simulation 5a, the oxygen limitation is acute and leads to the appearance of inert biomass. For simulation 2a, both NH_3_ and O_2_ assume the role of limiting substrate, for different areas of the biofilm.

**Figure 6:**
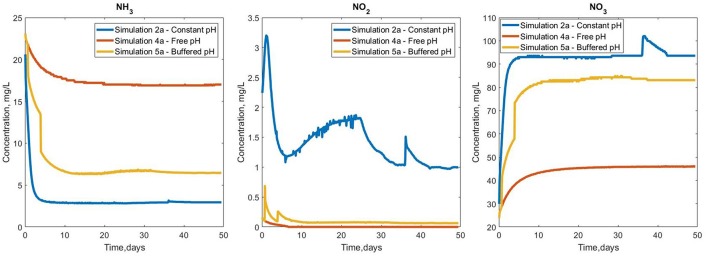
Bulk concentration profiles for ammonia, nitrite and nitrate for the three pH simulations.

The variations in overall biofilm growth (Figure S2) are reflected in the bulk concentration profiles for the soluble nutrients (Figure 6). The constant pH has the highest (> 90%) ammonia removal efficiency, which decreased to 77 and 43% in the case of in the buffered and free pH simulations, respectively. The production of nitrate and nitrite also seems to observe this trend, registering the lowest values for simulation 4a and mid-range concentrations for the buffered simulation 5a.

The buffered case (simulation 5a) shows decreases in total ammonia and spikes in the nitrate and nitrite bulk concentrations, as a result of pH correction events. There were small-scale oscillations (for example the NO_2_ and NO_3_ profiles in simulation 2a). These small oscillations are by-products of the numerical integration procedure and are too small to justify further refining of the implementation or time stepping.

### Anaerobic System

We also modeled a simple anaerobic ecosystem comprising glucose fermenters (using glucose as their substrate and producing acetate and hydrogen), acetoclastic methanogens (using the acetate to produce methane) and hydrogenotrophic methanogens (using hydrogen to produce methane).

The agents were seeded according to function, with the methanogens being placed next to the glucose fermenting agents, in an initial ratio of 1:1:1. In this way, each functional group was given equal access to its corresponding nutrients. The kinetic and thermodynamic parameters for the biological agents are presented in Table 3, together with the anabolic and catabolic reactions corresponding to each type.

**Table 3:** Kinetic and thermodynamic parameters for the biological agents in the anaerobic system.

**Anaerobic system**				
**Functional group**	**Kinetic parameters**			
	**q**_**max**_ **(mol** **·** **L**^**−1**^ **h**^**−1**^**)**	**K**_**s**_ **(mol** **·** **L**^**−1**^**)**	**k**_**decay**_ **(h**^**−1**^**)**	
**Glucose fermenter**	0.208	1.44_·_10^−3^	0.033	Batstone et al., 2006
Anabolic reaction	0.175 C_6_H_12_O_6_ + 0.2 NH_3_ → CH_1.8_O_0.5_N_0.2_ + 0.05 HCO_3_^**−**^+ 0.4 H_2_O + 0.05 H^+^			
Catabolic reaction	C_6_H_12_O_6_ + 4 H_2_O → 2 CH_3_COO^**−**^+ 2 HCO_3_^**−**^+ 4 H_2_ + 4 H^+^			
**Hydrogenmethanogen**	0.063	8.65_·_10^−4^	0.0125	Batstone et al., 2006
Anabolic reaction	HCO_3_^**−**^+ 0.2 NH_3_ + 2.1 H_2_ + H^+^ → CH_1.8_O_0.5_N_0.2_ + 2.5 H_2_O			
Catabolic reaction	0.25 HCO_3_^**−**^+ H_2_ + 0.25 H^+^ → 0.25 CH_4_ + 0.75 H_2_O			
**Acetatemethanogen**	0.100	5 · 10^−5^	0.0021	Batstone et al., 2006
Anabolic reaction	0.525 CH_3_COO^**−**^+ 0.2 NH_3_ + 0.475 H^+^ → CH_1.8_O_0.5_N_0.2_ + 0.4 H_2_O + 0.05 HCO_3_^**−**^			
Catabolic reaction	CH_3_COO^−^ + H_2_O → CH_4_ + HCO_3_^**−**^			
**Functional group**	**Thermodynamic parameters**			
	**ΔG formation (kJ/Cmole-X)**	**ΔG dissipation (kJ/C-mole-X)**	**Calculated yield (moleX/moleDonor)**	
Glucose fermenter	–67	236	0.656	Heijnen et al., 1992
Hydrogenmethanogen	–67	700	0.109	von Stockar, 2014
Acetatemethanogen	–67	500	0.064	von Stockar, 2014

Five conditions were simulated (Table 4):

- 1b “fixed boundary conditions simulation” where the top boundary conditions are fixed, the pH is also allowed to vary naturally
- 2b “dynamic boundary conditions” where the boundary conditions are variable and their values are computed using the reactor mass balance module
- 3b “fixed pH simulations” where the pH was kept constant in the entire computational domain
- 4b “de-coupled thermodynamics” where the yield coefficients are computed at the beginning of the simulation and they are assumed constant throughout; in this manner we do not make use of the thermodynamics module and decouple it
- 5b “coupled thermodynamics” where we compute the values of the yield coefficients function of the chemical species concentration for each bacterial agent in each grid cell of the simulation domain, coupling the thermodynamics module developed

**Table 4:** Initial simulation conditions for the anaerobic case study.

		**Anaerobic system**				
**Simulation conditions**		**Simulation #**				
		**1b**	**2b**	**3b**	**4b**	**5b**
**Concentration (mg/L)**		**Top boundary conditions**				
Glucose	94	Fixed	Variable	Fixed	Variable	Variable
NH_3_	1.7	Fixed	Variable	Fixed	Variable	Variable
CO_2_	4.4	Fixed	Fixed	Fixed	Fixed	Fixed
Acetate	0.6	Fixed	Variable	Fixed	Variable	Variable
H_2_	0.0013	Fixed	Variable	Fixed	Variable	Variable
CH_4_	0	Fixed	Variable	Fixed	Variable	Variable
**Initial value**		**pH Control**				
pH	7.5	Free	Free	Constant	Buffered	Buffered
		**Thermodynamics module**				
		Coupled	Coupled	Coupled	De-coupled	Coupled
		**Simulation descriptor**				
		Fixed BC/Free pH	Dynamic BC Free pH	Constant pH	De-coupled Thermodynamics	Coupled Thermodynamics

The initial concentrations for soluble species are adapted from Batstone et al. (2006) and Doloman et al. (2017), choosing a glucose concentration corresponding to 100 mg chemical oxygen demand (COD) per liter.

Very interestingly, though the overall biomass attained a steady state, the functional groups did not, even after 42 simulation days. We have therefore observed the effects of our five scenarios on the transient states in the first 1,000 h of this anaerobic community.

#### Reactor Coupling

**Figure 7:**
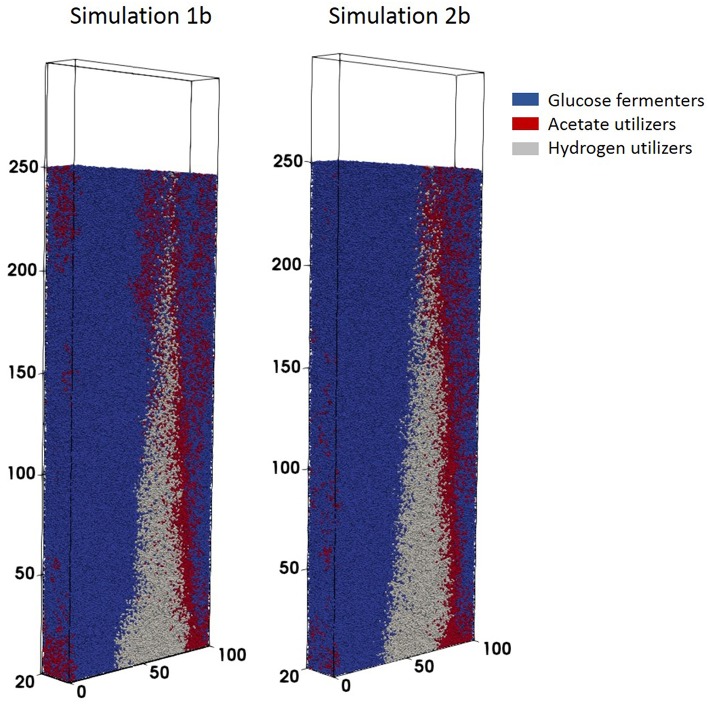
Biofilm structures obtained at *t* = 41.7 days for simulations 1b (fixed boundary conditions) and 2b (dynamic boundary conditions) for the anaerobic digestion system.

**Figure 8:**
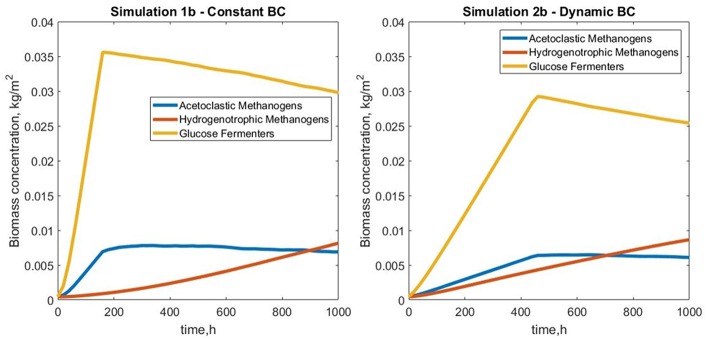
Total biomass concentration profiles for simulations 1b (fixed boundary conditions) and 2b (dynamic boundary conditions), showing the variations in bacterial species (glucose fermenters, acetate and hydrogen methanogens) concentrations vs. simulation time.

Simulation 1b (fixed boundary conditions) were subtly different from the dynamic simulation (2b), Figure 7. The biofilm growth rate was higher in the fixed conditions, reaching the imposed height in under 10 simulation days (Figure 8). The subsequent shearing of the top of the biofilm, lead to a decrease in the number of glucose fermenters, an increase in the hydrogen utilizers and a modest decrease in the acetate producers in both cases.

The biofilm profiles for the soluble species are also subtly different: acetate production is higher with fixed boundary conditions, with zones of high acetate concentration observed in both cases. The acetate hotspots coincided with the positions of high glucose fermenter activity and a paucity of acetogens.

**Figure 9:**
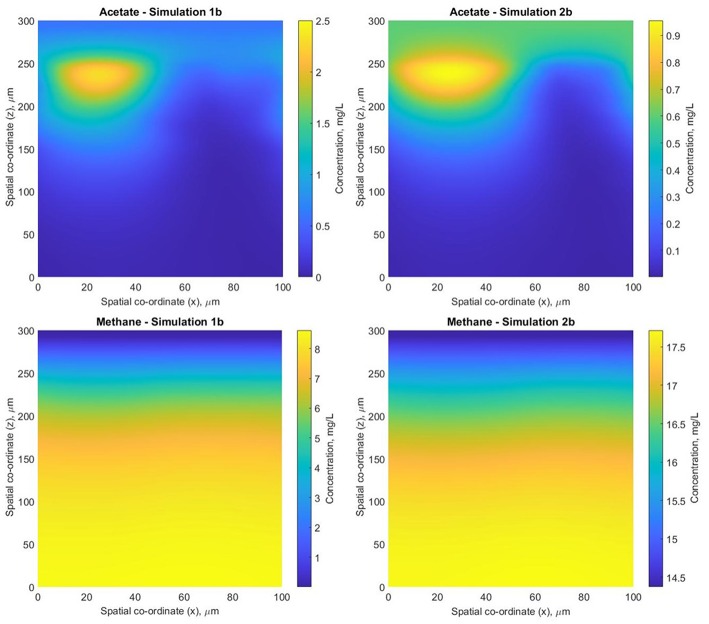
Biofilm acetate (CH_3_-COO^−^ and CH_3_-COOH) and methane (CH_4_) concentration profiles for simulations 1band 2b– 2D slices through the computational, normal to the substratum at width *y* = 10 μm inside the biofilm.

The conditions in 2b lead to a “healthier,” more productive ecosystem with more methane production, less acetate accumulation and a higher ratio of methanogens to fermenters. The higher levels of methane in the middle of the biofilm in simulation 2b tied in with the larger number of hydrogenotrophic methanogens (Figure 9).

#### Influence of pH

The importance of pH in anaerobic ecosystems is well-known (Lindner et al., 2015; Latif et al., 2017). Methanogenic species are affected in three important ways: pH affects free ammonia and ammonium ion concentrations and thus ammonia toxicity, pH values <5 are thought to be inhibitory and pH affects acetate speciation and thus the ecology of acetogenic methanogens. We have neglected ammonia and pH inhibition, the total ammonia concentration in the system is below that for inhibition threshold (0.05 to 1.5 gNH_3_-N/L) (Astals et al., 2018), the pH values never fell below 5 (even without buffering; Figure S3).

**Figure 10:**
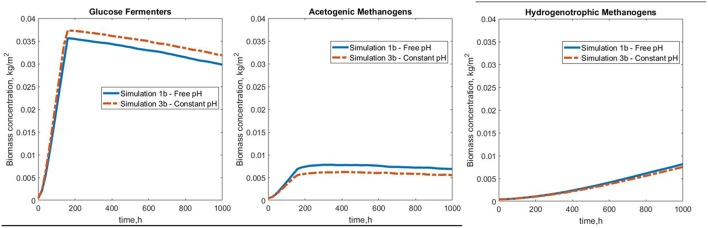
Biomass concentration profiles for simulations 1b (free pH) and 3b (constant pH) for the biofilm functional groups.

The results of the free and constant pH simulations (1b and 3b, respectively) show that the rate of biofilm formation and final total concentration of biomass were approximately the same in both scenarios. The lower availability of acetic acid at slightly basic pH lead to a lower overall concentration of acetoclastic methanogens when the pH is constant (simulation 3b). The glucose fermenters benefited from the constant pH levels (Figure 10).

The hydrogen and methane profiles are virtually identical in the two simulations, with a slight decrease in methane production for the case of simulation 3b (Figure S4).

#### Thermodynamics Considerations

For the anaerobic system, we also compared the outcome of considering fixed values for the yield coefficients (the standard approach in IbM; simulation 4b) vs. employing the thermodynamics module (simulation 5b). Both simulations employed pH buffering and dynamic boundary conditions.

**Figure 11:**
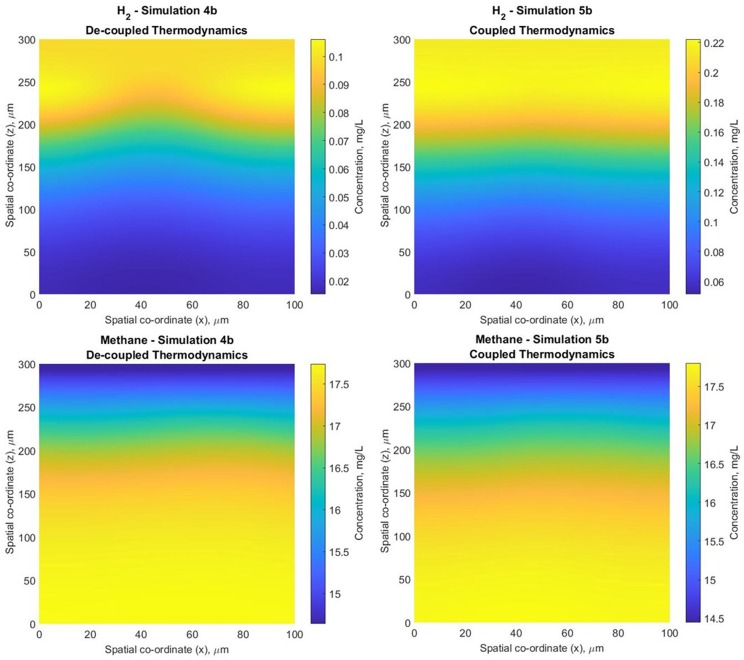
Hydrogen and methane biofilm concentration profiles for simulations 4b (constant biomass yield) and 5b (coupled thermodynamics module—biomass yield varies according to the available energy)−2D slices through the computational domain, normal to the substratum at width *y* = 10 μm.

The results are similar, but not identical. Coupled thermodynamics leads to fewer hydrogen utilisers (Figure S5) and thus greater hydrogen accumulation [though not to the point at which H_2_ becomes inhibitory—(Batstone et al., 2006)] and slightly lower methane production (in both biofilm and bulk) (Figure 11).

In the absence of experimental validation, it is difficult to say which approach produces better results. However, the fact that both simulations produce similar results is proof of the predictive capabilities of the thermodynamic approach, which can be employed for recently discovered bacterial species (e.g., complete ammonia oxidizers) or even hypothetical ones.

## Discussion

In this paper, we present the main functionalities of a new IbM framework, showcasing the impact of reactor coupling, pH variation and thermodynamic yield predictions on the growth of bacterial biofilms in aerobic and anaerobic conditions. This framework has certain important advantages.

Firstly, it can be applied to any ecological system for which we can determine the appropriate redox couples such as iron oxidation or sulfate reduction. The use of thermodynamics is a step toward “*ab initio*” modeling of microbial metabolisms in IbM that could be applied to almost any microbial system, as evidenced by our ability to model both a nitrifying and anaerobic systems. Such an approach could be very useful if we wished to know approximately how an unstudied, future or hypothetical community might behave. The next step would be to determine growth from first principles. Since growth is the yield multiplied by the substrate uptake rate, it could also be predicted relatively easily within this framework. However, we do not yet have the required predictive understanding of substrate uptake rate. We suspect that a predictive understanding of substrate uptake will emerge from the ongoing genomics revolution. The thermodynamic approach also requires us to specify which chemical components are taking part in microbial growth and to eschew the use of COD as a universal measure organic matter. Thus, the power of grounding a model in something as fundamental (and arguably infallible) as thermodynamics must be set against the limits to the number of species that can defined in a model (or validated in an experiment). However, we have not yet reached that limit. Despite the intrinsic power of a thermodynamics based approach, only one previous manuscript has even considered the use of this approach in individual based modeling (Araujo Granda et al., 2016). This work, in a simplified 2-dimensional model without speciation or pH, which are clear vital to a realistic evaluation of the ecological outcomes, has been mostly overlooked.

The inclusion of speciation and pH was our second step toward realism, and it is a powerful enabling feature of our thermodynamic approach (and by implication any putative *ab initio* future models). “Switching off” either the thermodynamic or the pH module gave a different outcome in the anaerobic module. Moreover, pH and speciation affected the availability of substrate and thus the microbial growth to a significant extent in the nitrification. We believe that an explicit pH submodule would enhance both more limited metabolic models, based solely on Monod kinetics, and more sophisticated models based on detailed metabolic models. For such a module permits us to consider the unavoidable effect of pH without invoking an empirical inhibition mechanism. The early work in this area demonstrated this point (Batstone et al., 2006), albeit with a fixed metabolism. Few have followed their lead (Doloman et al., 2017).

Our third important step toward realism was the incorporation of coupling, which is presenting the model as part of a larger community. Previous works have proposed a failure in coupling as an important fault of IbM. We are now able to confirm this supposition. The importance was evident in both models but was particularly profound in the context of nitrification where the nitrite oxidisers simply would not “grow” in an uncoupled system. We attribute our success in coupling to the use of small-time steps (<1 h) for the biological growth stages with a corresponding increase in computational requirements. Other computationally demanding steps toward realism include use of three dimensions, which in turn enabled the incorporation of computer intensive physical interactions. It was only possible to run such a model efficiently because of the substantial efforts we put into parallelising the code.

Nevertheless, it could be argued that at 5·10^5^ cells, the model is still not fast enough, and longer and larger simulations would have been more credible. In the future, we should aspire to around 10^8^ cells (± 300 μm × 1 mm^2^) (without the use of super-individuals) as this could be validated using microfluidics devices or microcosms from real systems. Ideally we would do this on a common, fast (parallelizable) and scalable modular platform analogous to LAMMPS but dedicated to microbial systems. Our colleagues have succeeded in modeling 2·10^7^ bacteria using the LAMMPS platform (Li et al., 2019) but without including pH and thermodynamics.

Those familiar with IbM will be aware that each of our “steps toward” realism have been made, albeit in isolation, before (Picioreanu et al., 2004, 2008; Araujo et al., 2015). We believe that our act of synthesis is also a form of innovation: it permits us to see the interaction (or lack thereof) between these features and is a useful step toward a day when IbM are not simply fascinating, but credible tools used in real world decision making. Making an IbM that would incorporate these fundamental features of a microbial community (growth, mechanics, chemistry and coupling) and yet still run relatively quickly on a normal HPC required inventive interdisciplinarity. The integration of innovation would also be easier if we shared a common, fast, scalable and modular platform for IbM destined for application.

## Data Availability

The source code and the simulations' set-up used to generate the data analyzed in this work are publicly available in Github, at https://github.com/nufeb/NUFEB/tree/master/examples/PAPER-NUFEB2. Animations for all the simulations presented are also available at this address.

## Author Contributions

VG and RG-C devised the study. VG analyzed and interpreted the data, and wrote the first draft of the manuscript. RG-C designed the implementation for the pH and thermodynamics modules. PJ designed the mechanical module implementation. BL and DT implemented the model in LAMMPS. DT parallelized the LAMMPS implementation. IO and TC provided over-all guidance of the work and editing of the text. All the authors contributed to the writing of the manuscript, revised it, and approved the final version.

### Conflict of Interest Statement

The authors declare that the research was conducted in the absence of any commercial or financial relationships that could be construed as a potential conflict of interest.
