# Supplementary material for: Individual Based Model Links Thermodynamics, Chemical Speciation and Environmental Conditions to Microbial Growth
Source: Front Microbiol. 2019 Aug 13;10:1871. doi: 10.3389/fmicb.2019.01871 (PMC6700366; doi:10.3389/fmicb.2019.01871)
Supplement: Supplementary file 1 [file Data_Sheet_1.docx]

Supplementary Material

1. Model parameters
   1. Chemical module

Table S1. Diffusion coefficients for the soluble compounds

| Chemical substance | Diffusion coefficients * 10^9^ m^2^ s^-1^ | Reference |
| --- | --- | --- |
| NH_3_ | 1.64 | Yaws (2009) |
| NO_2_ | 1.91 |  |
| NO_3_ | 1.9 |  |
| O_2_ | 2.1 |  |
| CO_2_ | 1.92 |  |
| H_2_ | 4.5 |  |
| C_6_H_12_O_6_ | 0.67 |  |
| CH_3_COOH | 1.29 |  |
| CH_4_ | 1.49 |  |
| NaOH | 1.33 |  |
| HCl | 1.4 |  |

Table S2. Dissociation reactions for the soluble compounds

| Chemical species | Dissociation reactions |
| --- | --- |
| NH_3_ | NH_3_ + H_2_O ↔ NH_4_^+^ + OH^-^ |
| NO_2_ | HNO_2_ + H_2_O ↔NO_2_^-^ + H_3_O^+^ |
| NO_3_ | HNO_3_ + H_2_O↔NO_3_^-^ + H_3_O^+^ |
| O_2_ | -- |
| CO_2_ | CO2 + H_2_O ↔H_2_CO_3_  H_2_CO_3_+ H_2_O ↔ HCO_3_^-^ + H_3_O^+^  HCO_3_^-^ + H_2_O ↔ CO_3_^2-^ + H_3_O^+^ |
| H_2_ | ----- |
| C_6_H_12_O_6_ | ---- |
| CH_3_COOH | CH_3_COOH + H_2_O ↔ CH_3_COO^-^ + H_3_O^+^ |
| CH_4_ | ---- |
| NaOH* | NaOH → Na^+^ + OH^-^ |
| HCl* | HCl → H^+^ + Cl^-^ |

The equilibrium constants ${(K}_{eq})$are computed using eq. (S1)

$K_{eq}=e^{-\frac{{\Delta G}_{dissociation}}{RT}}$ (S1)

where ${\Delta G}_{dissociation}$ is the free Gibbs energy corresponding to the dissociation reaction, computed using the standard free energies of formation, presented in Table S3, R is the ideal gas constant (kJ/mole/K) and T is the temperature (K).

Table S3. Gibbs free energy of formation for chemical compounds

| Chemical substance | Free Gibbs energy of formation (kJ/mole) | Reference |
| --- | --- | --- |
| NH_3_ | ΔG_f,_ _NH3_ = -26.57  ΔG_f,_ _NH4+_ = -79.37 | Perry and Green (2008) |
| NO_2_ | ΔG_f, HNO2_ = -50.60  ΔG_f,_ _NO2-_ = -32.20 |  |
| NO_3_ | ΔG_f, HNO3_ = -103.70  ΔG_f,_ _NO3-_ = -111.30 |  |
| CO_2_ | ΔG_f,_ _hydrolysis_ = -386.00  ΔG_f,_ _H2CO3_ = -623.16  ΔG_f,_ _HCO3-_ = -586.85  ΔG_f,_ _CO32-_ = -527.8 |  |
| CH_3_COOH | ΔG_f,_ _CH3COOH_ = -396.5  ΔG_f,_ _CH3COO-_ = -369.3 |  |

For the computation of the dissociation constants and the Gibbs energy of the anabolic and catabolic reactions the standard Gibbs energies listed in Table S3 are corrected for temperature, using eq. (S2).

$\Delta G_{r}=\Delta G_{r}^{o}+RTlnQ$ (S2)

where Q – reaction quotient; R – ideal gas constant, T – temperature;

1.1.4. Reactor specifications

Table S4. Design parameters for the bioreactor

| Parameter | Value | Reference |
| --- | --- | --- |
| Reactor volume | 1.25 L | Picioreanu et al. (2004) |
| Biofilm surface area | 0.1 m^2^ |  |
| Volume flow rate | 0.83 L h^-1^ |  |

- 1. Biological module

Table S5. Parameters for the biological agents

| Cell parameter | Value |
| --- | --- |
| density | 290 kg/m^3^ |
| division diameter | 1.25 µm |
| inert diameter | 0.58 µm |

- 1. Mechanical module

The friction force between two particles uses the already existing LAMMPS module, (<https://lammps.sandia.gov/doc/pair_gran.html> ). The parameters for cell-cell and cell–wall interactions are presented in Table S6.

Table S6. Cell – cell interaction parameters

| Parameter | | Cell – cell interactions | Cell – wall interactions |
| --- | --- | --- | --- |
| k_n_ | elastic constant for normal particle repulsion | 10^-4^ N/m | 2000 N/m |
| k_t_ | elastic constant for tangential contact | 2/7 * k_n_ | 2/7 * k_n_ |
| γ_n_ | damping coefficient in normal direction | 10^-5^ s^-1^ | 500 s^-1^ |
| γ_t_ | damping coefficient in tangential direction | ½ * γ_n_ | ½ * γ_n_ |
| γ_F_ | damping force coefficient | 10^-5^ s^-1^ | 1 s^-1^ |

1. Thermodynamic calculations

An example calculation for the yield of ammonia oxidizing micro-organisms is presented below.

1. catabolic reaction

For the catabolic reaction R_Cat_, we assume that the ammonia is oxidised to produce energy

NH_3_ + 1.5O_2_ → NO_2_^-^ + H_2_O + H^+^ R_Cat_

The free Gibbs energy of the catabolic reaction is thus, in standard conditions (assuming the reaction quotient is 1):

$\Delta G_{cat}=-267.4\frac{kJ}{mole {NH}_{3}}$

1. anabolic reaction

The half reaction for biomass formation:

0.2 NH_3_ + HCO_3_^-^ + 5.2H^+^ + 4.2e^-^ → CH_1.8_O_0.5_N_0.2_ + 2.5H_2_O R_1/2 – 1_

The half reaction for the electron donor (must supply the required electrons):

NH_3_ + 2H_2_O → NO_2_^-^ + 7H^+^ + 6e^-^ R_1/2 – 2_

The full anabolic reaction is assembled by combining the two reactions in such a manner that electrons are balanced between the two. Formally, we write this as:

R_Ana_ = R_1/2 – 1_ + 0.7 R_1/2 – 2_ (S3)

The full anabolic reaction R_Ana_ becomes

0.9 NH_3_ + HCO_3_^-^ + 0.3H^+^ → CH_1.8_O_0.5_N_0.2_ + 1.1 H_2_O + 0.7 NO^2-^  R_Ana_

The free Gibbs energy of the anabolic reaction is thus:

$\Delta G_{cat}=260.2\frac{kJ}{mole {NH}_{3}}$

Because both ammonia and nitrite oxidisers are autotrophic species and their metabolism involves reverse electron transfer, for the dissipation energy we used the value of 3500 kJ/mole, proposed by (Heijnen et al., 1992).

We can now compute the maximum growth yield by combining the catabolic, anabolic and dissipation free energies

$Y_{XS}=\frac{{\Delta G}_{cat}}{{\Delta G}_{ana}+{\Delta G}_{dis}}$ = 0.067 C-mole biomass/ mole NH_3_

The overall growth reaction (R_OVG_) is obtained by running the catabolism λ (1/Y_XS_) times and adding it to the anabolism:

R_OVG_ = R_Ana_ + λ R_Cat_ (S4)

15.86 NH_3_ + HCO_3_^-^ + 22.46O_2_→ CH_1.8_O_0.5_N_0.2_ + 15.96 H_2_O + 15.66NO^2-^ + 14.66H^+^ R_OVG_

1. Supplementary Results
   1. Nitrification
      1. Reactor coupling

The effect of using fixed top boundary conditions vs. updating them function of the biofilm activity is apparent from Fig. S1. The biomass concentration profiles differ significantly between the two cases, showing a thriving active biomass population in case of the latter simulation.


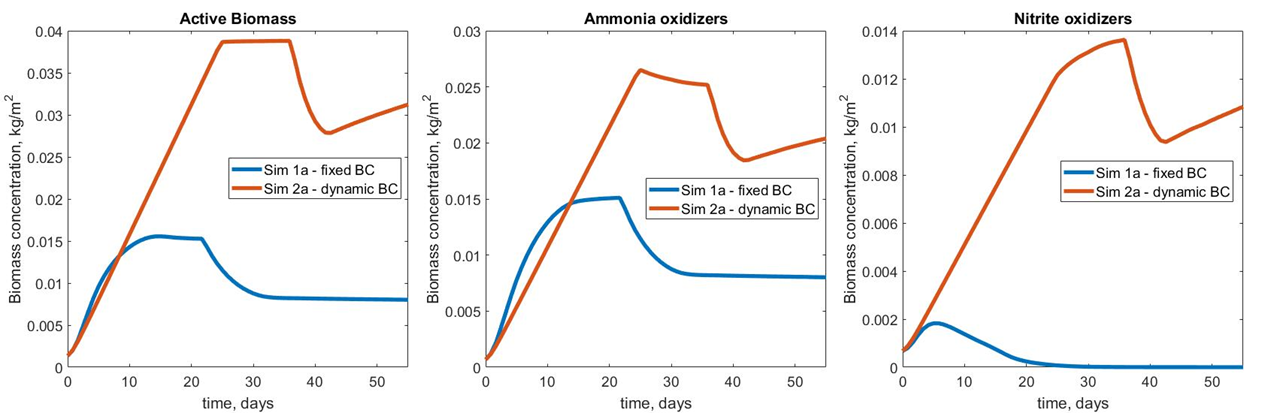


Figure S1. Biomass concentrations vs. time for simulation 1a and 2a

- - 1. pH influence

The pH has a strong influence on the overall growth rate of the biofilm and a less pronounced one on the biofilm composition. The three systems are reaching the 250 µm-imposed height at different time steps, while the biomass concentration and the ratio between the two agent types were similar for all cases.


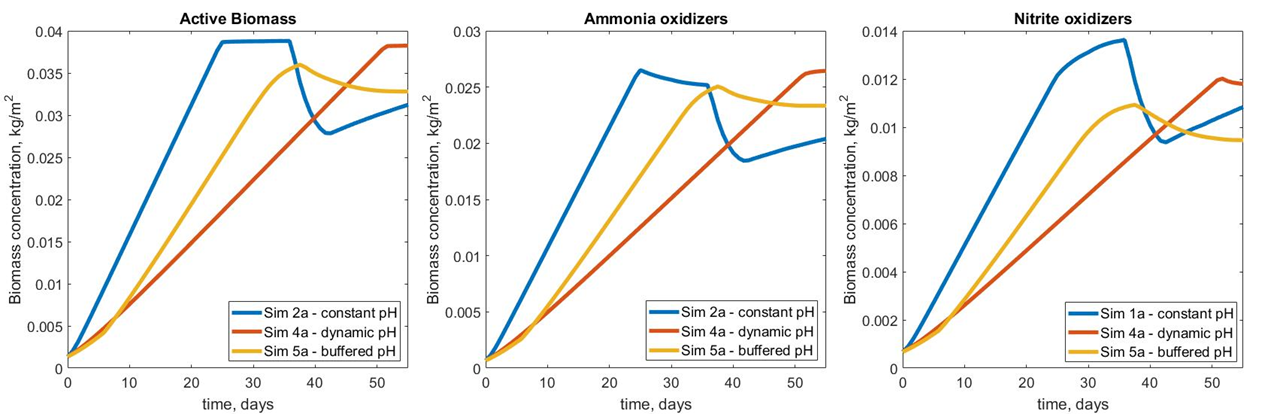


Figure S2. Biomass concentrations vs. time for simulation 2a, 4a and 5a

- 1. Anaerobic digestion
     1. pH influence

Unlike the nitrification case, the pH control strategy (i.e. allowing the pH to vary naturally and attempting to maintain a constant pH in the biofilm – Fig. S3) does not produce large differences in biomass concentrations or soluble species profiles.


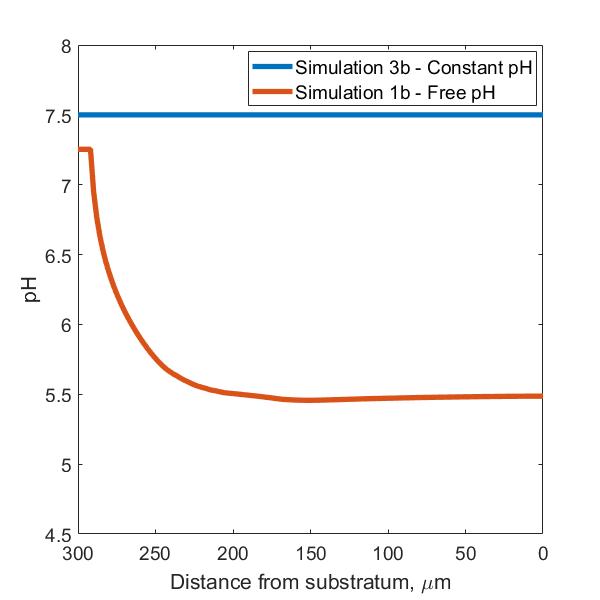


Figure S3. pH profiles – in the Oz direction at coordinates x = 50 µm and y = 10 µm inside the anaerobic biofilm

The soluble species concentrations differ subtly between the two simulations; Fig. S4 presents the percentage variation, computed using eq. (S5), between them.

${variance}_{\%}=\frac{S_{x,y,z}^{Sim 1b}-S_{x,y,z}^{Sim 3b}}{S_{x,y,z}^{Sim 1b}}\cdot100$ (S5)


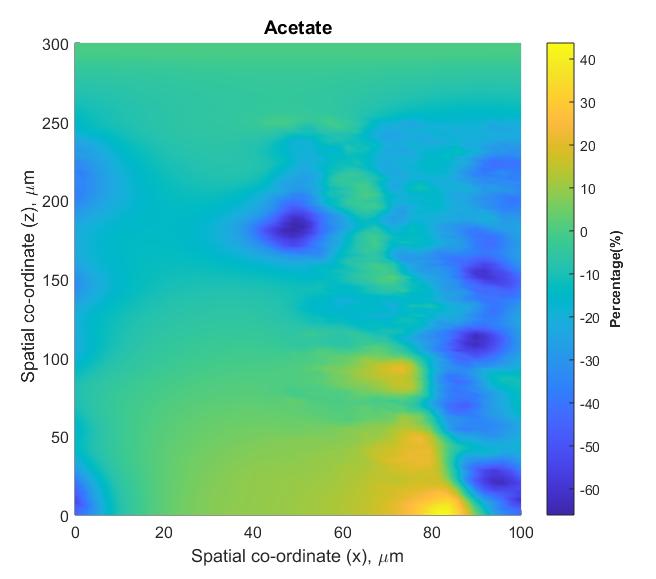

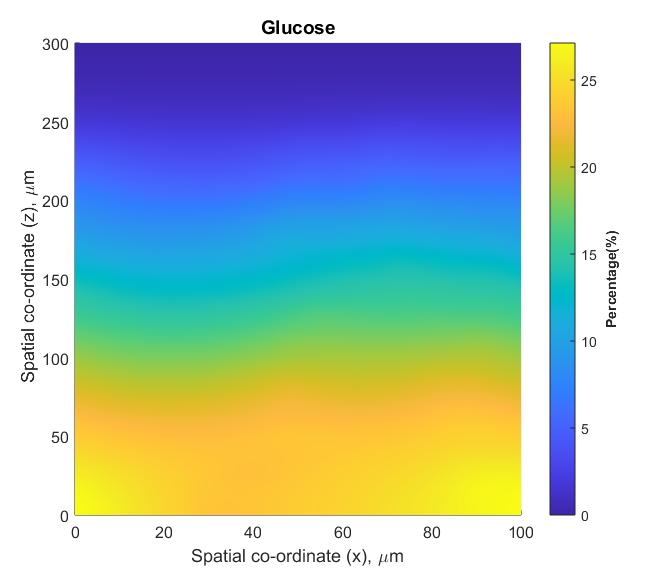

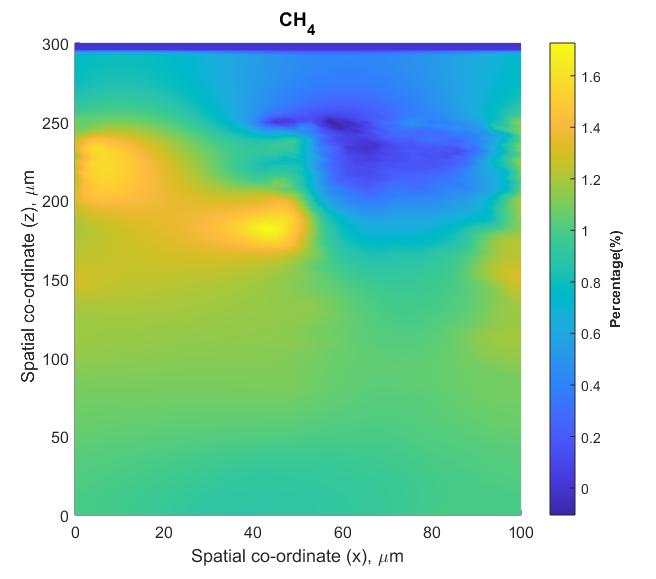

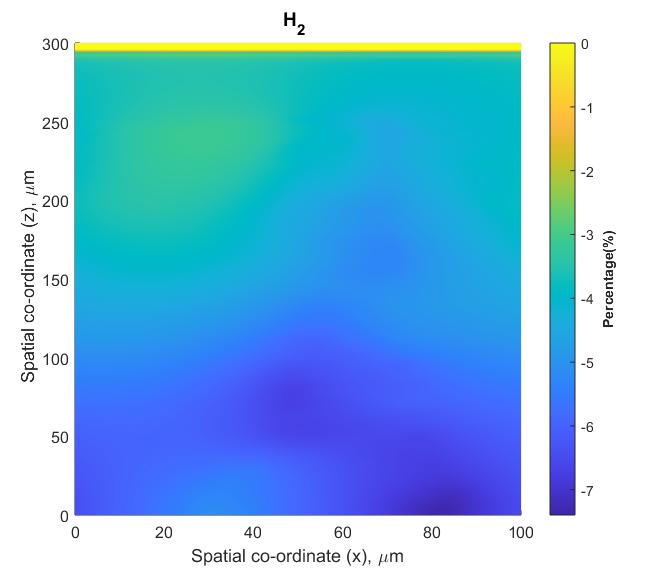


Figure S4. The percent variance for glucose, acetate, hydrogen and methane for simulations 1b (free-varying pH) and 3b (constant pH) at t = 1000 h, as 2D slices through the computational domain, normal to the substratum at width y = 10 µm

- - 1. Thermodynamics influence

For the simulations performed with (5b) and without (4b) the thermodynamics module, the total active biomass concentration was similar (Fig. S5). The fixed yield biofilm (4b) is richer in methanogens, having twice as many hydrogen utilisers compared to simulation 5b and approximately 25% less acetoclastic methanogens.


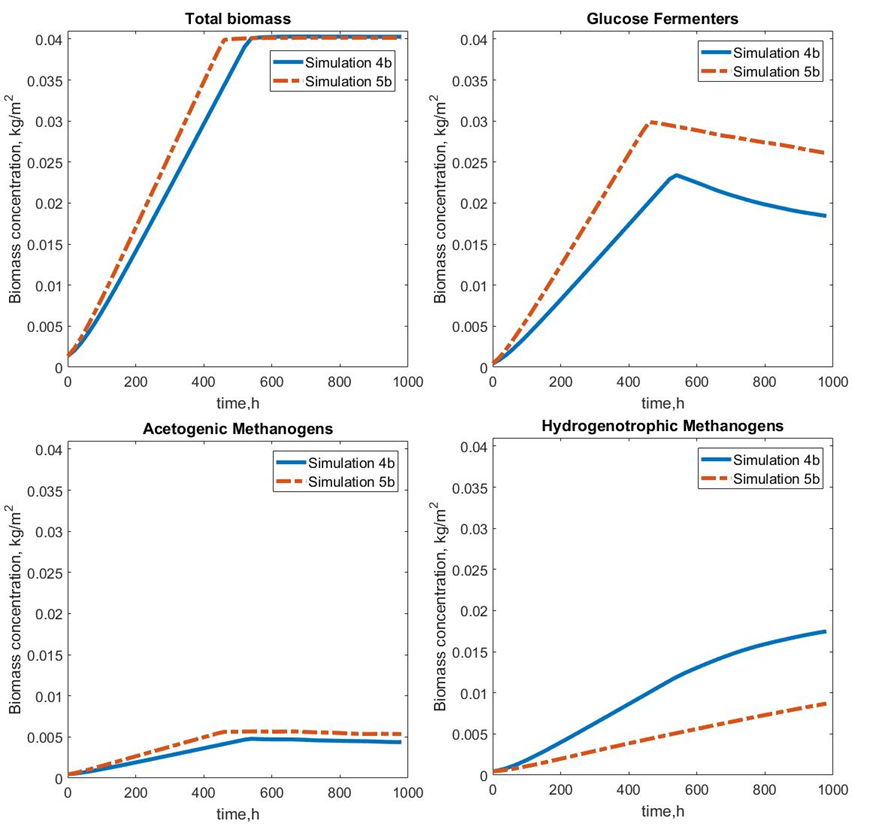


Figure S5. The concentration profiles for total biomass and each bacterial species present in the biofilm for simulations performed with (simulation 5b) and without (simulation4b) the thermodynamics module

References

Heijnen, J. J., Loosdrecht, M. C. M. v. and Tijhuis, L. (1992). "A Black Box Mathematical Model to Calculate Auto- and Heterotrophic Biomass Yields Based on Gibbs Energy Dissipation." Biotechnology and Bioengineering **40**(1992): 1139-1154.

Perry, R. H. and Green, D. W. (2008). Perry's chemical engineers' handbook. New York, New York : McGraw-Hill.

Picioreanu, C., Kreft, J. U. and van Loosdrecht, M. C. M. (2004). "Particle-based multidimensional multispecies Biofilm model." Applied and Environmental Microbiology **70**(5): 3024-3040.

Yaws, C. L. (2009). Transport Properties of Chemicals and Hydrocarbons. Boston, William Andrew Publishing.
